# Antibiotic resistance alters the ability of *Pseudomonas aeruginosa* to invade bacteria from the respiratory microbiome

**DOI:** 10.1093/evlett/qrae030

**Published:** 2024-06-30

**Authors:** Selina Lindon, Sarah Shah, Danna R Gifford, Cédric Lood, Maria A Gomis Font, Divjot Kaur, Antonio Oliver, R Craig MacLean, Rachel M Wheatley

**Affiliations:** Department of Biology, University of Oxford, Oxford, United Kingdom; Department of Biology, University of Oxford, Oxford, United Kingdom; Division of Evolution, Infection and Genomics, School of Biological Sciences, The University of Manchester, Manchester, United Kingdom; Department of Biology, University of Oxford, Oxford, United Kingdom; Servicio de Microbiología, Hospital Universitari Son Espases, Instituto de Investigación Sanitaria Illes Balears (IdISBa), CIBERINFEC, Palma de Mallorca, Spain; Department of Biology, University of Oxford, Oxford, United Kingdom; Servicio de Microbiología, Hospital Universitari Son Espases, Instituto de Investigación Sanitaria Illes Balears (IdISBa), CIBERINFEC, Palma de Mallorca, Spain; Department of Biology, University of Oxford, Oxford, United Kingdom; Department of Biology, University of Oxford, Oxford, United Kingdom; School of Biological Sciences, Queen’s University Belfast, Belfast, United Kingdom

**Keywords:** antibiotic resistance, microbiome, *Pseudomonas aeruginosa*, microbial interactions

## Abstract

The emergence and spread of antibiotic resistance in bacterial pathogens is a global health threat. One important unanswered question is how antibiotic resistance influences the ability of a pathogen to invade the host-associated microbiome. Here we investigate how antibiotic resistance impacts the ability of a bacterial pathogen to invade bacteria from the microbiome, using the opportunistic bacterial pathogen *Pseudomonas aeruginosa* and the respiratory microbiome as our model system. We measure the ability of *P. aeruginosa* spontaneous antibiotic-resistant mutants to invade pre-established cultures of commensal respiratory microbes in an assay that allows us to link specific resistance mutations with changes in invasion ability. While commensal respiratory microbes tend to provide some degree of resistance to *P. aeruginosa* invasion, antibiotic resistance is a double-edged sword that can either help or hinder the ability of *P. aeruginosa* to invade. The directionality of this help or hindrance depends on both *P. aeruginosa* genotype and respiratory microbe identity. Specific resistance mutations in genes involved in multidrug efflux pump regulation are shown to facilitate the invasion of *P. aeruginosa* into *Staphylococcus lugdunensis*, yet impair invasion into *Rothia mucilaginosa* and *Staphylococcus epidermidis*. *Streptococcus* species provide the strongest resistance to *P. aeruginosa* invasion, and this is maintained regardless of antibiotic resistance genotype. Our study demonstrates how the cost of mutations that provide enhanced antibiotic resistance in *P. aeruginosa* can crucially depend on community context. We suggest that attempts to manipulate the microbiome should focus on promoting the growth of commensals that can increase the fitness costs associated with antibiotic resistance and provide robust inhibition of both wildtype and antibiotic-resistant pathogen strains.

## Introduction

Antibiotic resistance in pathogenic bacteria has emerged as a serious threat to public health (Ikuta et al., 2022; Murray et al., 2022). Infections caused by antibiotic-resistant pathogens are associated with worse clinical outcomes for patients, longer hospitalizations, and higher healthcare costs (Friedman et al., 2016). The microbiome can provide protection against colonization by pathogenic bacteria. This microbiome-mediated colonization resistance can be conferred through a variety of mechanisms, including induction of host immune responses, metabolic niche exclusion, and direct antagonistic interactions such as toxin production (Caballero-Flores et al., 2023).

One key unresolved question is how antibiotic resistance impacts the ability of pathogens to colonize hosts. Antibiotic resistance is often associated with fitness costs (Andersson & Hughes, 2010; MacLean & San Millan, 2019; Vogwill & MacLean, 2015), and work by others has demonstrated that presence of a microbial community can increase the fitness costs associated with resistance (Baumgartner et al., 2020; Klümper et al., 2019), suggesting that resistant pathogens should have a decreased ability to invade host-associated microbiomes. Yet, many antibiotics were originally isolated from microbes or are modifications of microbial products and mediate inter-bacterial competition in their natural capacity (Clardy et al., 2009). As such, mechanisms of antibiotic resistance may also provide cross-resistance to anticompetitor toxins produced by commensal microbes in the microbiome. In these scenarios, resistant pathogens may have an increased ability to invade the microbiome, but it is unknown how these factors trade off against each other. To fully comprehend the consequences of antibiotic resistance, we need to understand how antibiotic resistance impacts the ability of a bacterial pathogen to interact with the microbes in its surroundings and ultimately its ability to invade the microbiome. This has clear significance for understanding the potential of resistant strains to transmit between patients.

Here we set out to investigate how resistance alters the invasion ability of a pathogen, using the opportunistic bacterial pathogen *Pseudomonas aeruginosa* and the respiratory microbiome as our model system. *Pseudomonas aeruginosa* can cause a wide range of infections but is particularly problematic in the lungs. *Pseudomonas aeruginosa* is a major causative pathogen in serious short-term infections such as ventilator-associated pneumonia and is able to colonize the lungs of patients with bronchiectasis or cystic fibrosis, resulting in long-term recurring infections. In addition to widespread multidrug resistance (Oliver et al., 2015), *P. aeruginosa* is also known for its impressive arsenal of virulence factors and anticompetitor weaponry (Granato et al., 2019). All of this helps *P. aeruginosa* function as a powerful invasive species that can cause difficult-to-treat infections associated with a high burden of mortality (Murray et al., 2022). The respiratory microbiome can provide protection against pathogen colonization (reviewed here: Dickson & Huffnagle, 2015; Dickson et al., 2016; Man et al., 2017; Natalini et al., 2023; Yagi et al., 2021). In healthy individuals, common constituent members of the respiratory microbiome include *Streptococcus*, *Staphylococcus*, *Prevotella*, *Veillonella*, and *Rothia* species (Dickson et al., 2016; Narendrakumar & Ray, 2022), and the lung microbiota largely resembles the upper respiratory tract microbiota, but at lower densities (Bassis et al., 2015; Marsh et al., 2016).

In this study, we generated spontaneous resistant mutants of *P. aeruginosa* to three clinically important antipseudomonal drugs (ciprofloxacin, ceftazidime, and meropenem). Both meropenem and ceftazidime target cell wall synthesis, whereas ciprofloxacin targets nucleic acid synthesis via DNA gyrase (Kapoor et al., 2017). We tested the ability of these mutants to invade pre-established cultures of six commensal respiratory microbiome strains. This generated an assay in which specific resistance mutations could be linked to changes in invasion ability. While these respiratory microbes tended to provide resistance to *P. aeruginosa* invasion, we found cases where antibiotic resistance could help or hinder the ability of *P. aeruginosa* to invade, and the directionality of this was dependent on both *P. aeruginosa* genotype and respiratory microbe identity. The fitness costs associated with antibiotic resistance are context dependent (Baumgartner et al., 2020; Klümper et al., 2019; Santos-Lopez et al., 2019), and here we demonstrate how the cost of resistance mutations in *P. aeruginosa* can change depending on the presence or absence of bacteria from the respiratory microbiome.

## Methods

### Respiratory microbiome strains

The six respiratory microbiome species used were: *Staphylococcus epidermidis*, *Rothia mucilaginosa*, *Streptococcus gordonii*, *Staphylococcus lugdunensis*, *Streptococcus oralis*, and *Streptococcus salivarius* (Supplementary Table S1). These microbiome species were selected due to presence and relevance in the respiratory microbiome, availability of strain, and ability of strain to grow in proposed assay conditions.

### Generation of antibiotic-resistant *P. aeruginosa* strains

A green fluorescent protein (GFP)-tagged strain of *P. aeruginosa* PAO1 (PAO1-GFP; Vogwill et al., 2016) was used so that reads in the GFP channel could measure *P. aeruginosa* invasion ability (excitation: 485/20, emission: 516/20). This strain was created by insertion of GFP and a gentamicin resistance cassette into the Tn7 transposon insertion site of the reference strain of *P. aeruginosa* PAO1 (Vogwill et al., 2016). Three independent spontaneous resistant mutants were selected on each of the three antibiotics (ciprofloxacin, ceftazidime, and meropenem) at the clinical breakpoints according to the EUCAST Clinical Breakpoint Tables v.12.0 (ciprofloxacin 0.5 μg/ml, ceftazidime 8 μg/ml, and meropenem 8 μg/ml) (EUCAST, 2024). These antibiotics were chosen to represent different classes of clinically important antipseudomonal drugs for treating *P. aeruginosa* infections (fluoroquinolones, cephalosporins, and carbapenems).

To generate spontaneous resistant mutants, PAO1-GFP was grown from glycerol stock on LB (Lysogeny Broth) Miller with agar (Sigma-Aldrich) plates supplemented with 15 μg/ml gentamicin overnight at 37 °C. Single independent colonies were grown in LB Miller broth overnight at 37 °C in biological triplicate with shaking at 225 rpm, then 150 ul of overnight culture was plated on LB Miller agar plates supplemented with either 0.5 μg/ml ciprofloxacin, 8 μg/ml ceftazidime, or 8 μg/ml meropenem. Spontaneous resistant mutants of PAO1-GFP successfully grew on both 0.5 μg/ml ciprofloxacin and 8 μg/ml ceftazidime. A single colony from each plate was inoculated into LB Miller broth supplemented with the corresponding antibiotic at 50% of the selective concentration (i.e., 0.25 μg/ml ciprofloxacin and 4 μg/ml ceftazidime) for overnight growth. Three independent resistant mutants for each antibiotic were stored as glycerol stocks at −80 °C (Supplementary Table S1). No resistant mutants of PAO1-GFP were able to grow on meropenem 8 μg/ml, and so PAO1-GFP was serially passaged through progressively higher meropenem concentrations over 3 days to generate meropenem-resistant mutants, ramping from 1 μg/ml to 2 μg/ml to 4 μg/ml with transfer every 24 hr before plating on 8 μg/ml meropenem. The same procedure for stocking was then followed as above. A total of nine resistant mutants of PAO1-GFP were generated across the three antibiotics which are hereby referred to as: ciprofloxacin-resistant mutants 1–3 (cipR1-cipR3), ceftazidime-resistant mutants 1–3 (cefR1-cefR3), and meropenem-resistant mutants 1–3 (merR1-merR3).

### Antibiotic resistance phenotyping via minimum inhibitory concentration assays

Isolates were grown from glycerol stocks on LB Miller agar plates overnight at 37 °C. Single colonies were then inoculated into tryptic soy broth (Sigma-Aldrich) for overnight growth at 37 °C with shaking at 225 rpm, after which overnight suspensions were serial diluted to ~5 × 10^5^ CFU/ml. Antibiotic resistance phenotyping was carried out as minimum inhibitory concentration (MIC) assays via broth microdilution as defined by EUCAST guidelines (EUCAST, 2022), with the alteration of tryptic soy broth for the growth media to replicate the media used for the invasion assays. Minimum inhibitory concentration was calculated using twofold dilution series for meropenem (from 0.5 to 64 μg/ml), ciprofloxacin (from 0.03125 to 4 μg/ml), and ceftazidime (from 0.5 to 64 μg/ml) designed to capture ranges either side of the clinical breakpoint (EUCAST, 2024). Growth inhibition was defined as OD_595_ < 0.200 and we calculated the MIC of each isolate as the median MIC score from three biological independent assays of each isolate. For some strains, the MIC was the lowest concentration in this range. However, as we were using this assay to determine increased resistance around the clinical breakpoint that sat in the midpoint of these ranges, this was not an issue.

### Growth rate assays

Growth rate assays were used to characterize growth of the antibiotic-resistant mutants compared to the ancestral strain (PAO1-GFP) in tryptic soy broth in the absence of any respiratory microbes. *Pseudomonas aeruginosa* strains were grown from glycerol stock on LB Miller agar plates overnight at 37 °C, then inoculated into tryptic soy broth for 18–20 hr overnight growth at 37 °C with shaking at 225 rpm. Overnight suspensions were diluted to an OD_595_ of ~0.05 and placed within the inner 60 wells of a 96-well plate equipped with a lid. To assess growth, isolates were grown in tryptic soy broth at 37 °C in a BioTek Synergy 2 microplate reader set to moderate continuous shaking for 24 hr and reads in the GFP channel (excitation: 485/20, emission: 516/20) were taken at 10-min intervals. Relative fluorescence units (RFU) of *P. aeruginosa* growth over 24 hr were plotted using the Growthcurver package in R (Sprouffske & Wagner, 2016).

### Invasion assays of GFP-tagged *P. aeruginosa* strains into the respiratory microbiome strains

An invasion assay in which endpoint measurements of fluorescence in the GFP channel (excitation: 485/20, emission: 516/20) at 24 hr was used to measure the ability of PAO1-GFP and the nine spontaneous antibiotic-resistant mutants to invade dense overnight cultures of the six respiratory microbiome strains. To conduct this assay, the 10 *P. aeruginosa* strains (PAO1-GFP, cipR1, cipR2, cipR3, cefR1, cefR2, cefR3, merR1, merR2, and merR3) and six respiratory microbiome strains (*S. epidermidis, R. mucilaginosa, S. gordonii, S. lugdunensis, S. oralis*, and *S. salivarius*) were grown from glycerol stock on LB Miller agar plates overnight at 37 °C. Single colonies of each *P. aeruginosa* strain were inoculated into the inner 60 wells of a 96-well plate with wells filled with 200 ul tryptic soy broth for overnight growth at 37 °C and shaking at 225 rpm, for six biological replicates per strain. Single colonies of each respiratory microbiome strain were inoculated into 14 ml falcon tubes filled with 7 ml tryptic soy broth for overnight growth at 37 °C with shaking at 225 rpm, for six biological replicates per strain.

The next day, a black-sided 96-well plate was labeled for each respiratory microbiome strain, and the following plate layout was used to arrange respiratory microbe invasions (Supplementary Figure S1). In brief, each plate contained six biological replicates of a respiratory microbiome strain that had been allowed to grow for 24 hr. Six biological replicates of the 10 *P. aeruginosa* strains were then inoculated into the wells containing the pre-established respiratory microbe cultures, with the invasion set up as a 1/20 dilution of *P. aeruginosa*. Three 96-well plates were used for tryptic soy broth controls, in which the same six replicates of the 10 *P. aeruginosa* strains were inoculated (1/20 dilution) into sterile tryptic soy broth for three technical replicates of *P. aeruginosa* growth. This paired growth of *P. aeruginosa* invasion inoculations was done in order to calculate *P. aeruginosa* invasion into respiratory microbe culture as a percentage of the growth achieved in rich media in the absence of that respiratory microbe. The plates were incubated for 24 hr at 37 °C with shaking at 225 rpm. After 24 hr, OD_595_ and GFP (excitation: 485/20, emission: 516/20) of the respiratory microbiome invasions and the tryptic soy broth paired growth assays were read in a BioTek Synergy 2 microplate reader.

Plates in which PAO1-GFP did not grow in 24 hr were then incubated for a further 12 hr, and OD_595_ and GFP were measured again at 36 hr. Invasion ability is given as percentage RFU (arbitrary unit) of the invasion assay standardized against RFU in the absence of the respiratory microbe. Fluorescence is proportional to cell density across the range used in our experiments. Prior to these invasion assays in which endpoint measurements were taken, invasion was first continually monitored in a BioTek Synergy 2 microplate reader taking reads at 10-min intervals for 24 hr. This preliminary data were used to inform the endpoint assays and produce invasion growth curves for illustrative purposes (Supplementary Figure S2), which was done using the Growthcurver package in R (Sprouffske & Wagner, 2016).

### Genome sequencing and variant detection of spontaneous antibiotic-resistant mutants of *P. aeruginosa*

The ten *P. aeruginosa* strains (PAO1-GFP, cipR1, cipR2, cipR3, cefR1, cefR2, cefR3, merR1, merR2, and merR3) were sequenced using Illumina short-read sequencing. DNA extraction, library preparation, and sequencing were performed by MicrobesNG (Birmingham, UK) according to their protocols. Briefly, ~4–6 × 10^9^ cells were concentrated and suspended in 500 μl of DNA/RNA Shield (Zymo Research) in 2 ml screw cap tubes, and stored at room temperature prior to sample submission. Between 5 and 40 μl of cell suspension was lysed with 120 μl TE buffer containing lysozyme (final concentration 0.1 mg/ml) and RNase A at 37 °C for 25 min. Protein digestion was performed using Proteinase K and SDS with incubation at 65 °C for 5 min. Genomic DNA was purified using an equal volume of paramagnetic beads (SPRI beads, Beckman Coulter) and resuspended in EB buffer (10mM Tris–Hcl, pH 8.0). DNA was quantified with QuantiT dsDNA HS Kit (ThermoFisher Scientific) using an Eppendorf AF2200 plate reader and diluted to an appropriate concentration for library preparation.

Library preparation was performed using the Nextera XT Library Prep Kit according to the manufacturer’s protocol with modifications (i.e., twofold increase in input DNA, PCR elongation increased to 45 s). DNA quantification and library preparation were performed using a Hamilton Microlab STAR liquid handling system. Libraries were sequenced using the Illumina NovaSeq6000 platform using a 250 bp paired-end protocol. Read adapter trimming was performed using Trimmomatic version 0.30 (Bolger et al., 2014) with a sliding window quality cutoff of Q15. Trimmed reads were aligned to the PAO1-UW reference genome (NCBI RefSeq accession NC_002516.2) (Klockgether et al., 2010). Variants in PAO1-GFP and the resistant strains were called using the breseq 0.36.1 pipeline (Deatherage & Barrick, 2014). Variants reported in the main text are those found in the resistant strains compared to the PAO1-GFP background and called at 100% (Supplementary Table S2). Gene descriptions in Supplementary Table S2 are added from the *Pseudomonas* genome database (Winsor et al., 2016).

### Spent media assays with *S. lugdunensis*

To better characterize the interaction between *P. aeruginosa* and *S. lugdunensis*, we repeated the invasion assays using clinical isolates of *P. aeruginosa* from two patients where meropenem resistance evolved during infection and the de novo resistance mutations are known (Supplementary Table S1; Wheatley et al., 2021, 2022). A spent media assay was used for this invasion assay as the clinical isolates were not GFP-tagged (Dos Santos et al., 2022). The invasion assay method used previously was conducted with the following alterations. After overnight growth of *S. lugdunensis* cultures, spent media was prepared via centrifugation at 16,000 g/min for 1 min to pellet the *S. lugdunensis* cells followed by filter sterilization of the supernatant through a 0.22 μM filter. *Pseudomonas aeruginosa* strains were inoculated into this cell-free spent media (1/20 dilution) in a 96-well plate as described above alongside paired replicate inoculations into sterile tryptic soy broth. For each clinical *P. aeruginosa* genotype (ST782-WT, *oprD mexR* ST782, ST17-WT, and *oprD* ST17), three biological replicates of three genetically identical isolates from the patient were measured (Supplementary Table S1), and PAO1-GFP was included in these assays as a control.

To test for the presence of potential growth inhibition molecules in the supernatant of *S. lugdunensis*, we prepared fresh cell-free spent media as described above. The media was subsequently used to either (a) spot 10 µl directly onto, or (b) inoculate a sterile filter disc placed onto, plates where PAO1-GFP had been spread using sterile beads. These plates were inoculated with dilutions of PAO1-GFP and we selected the dilution that resulted in confluent CFU growth. This was done in triplicate, using ddH_2_O as negative control. Plates were incubated overnight and inspected for the presence of zones of inhibition.

### Statistical analysis

Statistical analysis was done in R Studio Version 1.1.463 (RStudio Team, 2020). Differences in invasion ability between the *P. aeruginosa* antibiotic-resistant strains and PAO1-GFP were assessed using a one-way ANOVA followed by a post hoc Dunnett’s test. The *DescTools R* package was used to implement the Dunnett’s test (Signorell et al., 2019) and significance was determined at *p* < 0.05. One tailed unpaired *t*-tests were used to test for differences in growth in the *S. lugdunensis* spent media assays and significance was determined at *p* < 0.05.

## Results

### Characterization of *P. aeruginosa-*resistant mutants

**Figure 1.**
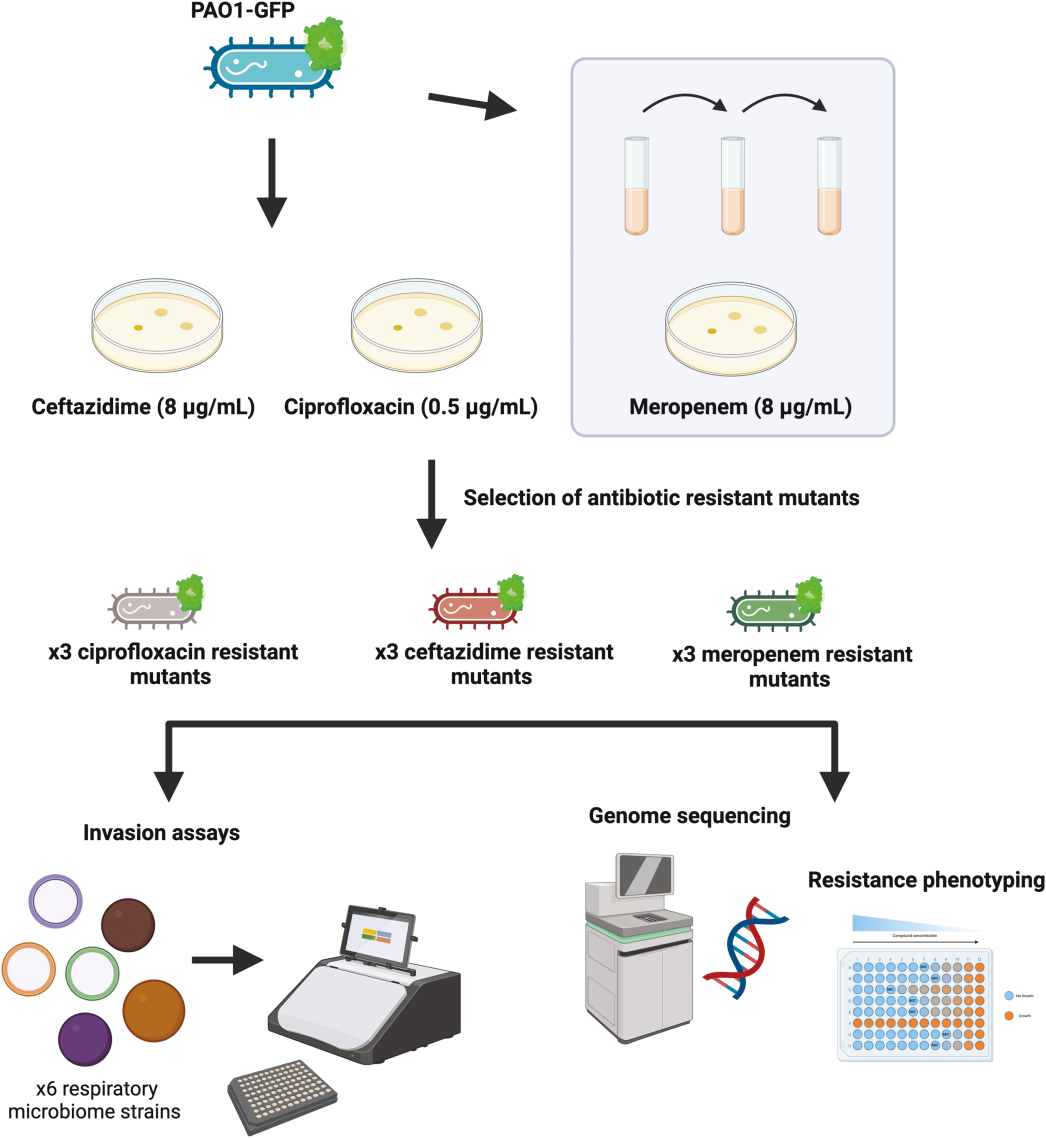
Schematic for selection of *P. aeruginosa* antibiotic-resistant mutants, subsequent strain characterization (via genome sequencing and resistance phenotyping), and use of strains in invasion assays.

Spontaneous resistant mutants of GFP-tagged *P. aeruginosa* (PAO1-GFP) were selected at the clinical breakpoints for ciprofloxacin (cipR1-cipR3), ceftazidime (cefR1-cefR3) and meropenem (merR1-merR3) to generate nine unique resistant mutants in total (Figure 1). Resistance phenotyping, genome sequencing, and growth rate assays were carried out to characterize these resistant mutants (Table 1; Supplementary Table S2). Selection on ciprofloxacin was associated with the acquisition of mutations in *nfxB*, which encodes NfxB, a transcriptional repressor of the *mexCD*-*oprJ* operon (Purssell & Poole, 2013). Mutations in *nfxB* upregulate the expression of the multidrug efflux pump MexCD-OprJ (Pang et al., 2019; Poole et al., 1996). Efflux pump overexpression is typically associated with a multidrug resistance phenotype, but the *nfxB* mutations were only associated with increases in resistance to ciprofloxacin (Table 1). One ciprofloxacin-resistant mutant (cipR2) had a deletion that included both *nfxB* and *morA*, predicted to encode a motility regulator involved in flagellar development and biofilm formation (Choy et al., 2004).

**Table 1.** Characterization of PAO1-GFP and resistant mutants (cipR1, cipR2, cipR3, cefR1, cefR2, cefR3, merR1, merR2, and merR3).

Strain	Ciprofloxacin MIC (μg/ml)	Ceftazidime MIC (μg/ml)	Meropenem MIC (μg/ml)	Mutated gene(s)	Mutation type
PAO1-GFP	0.25	0.5	0.5		
cipR1	4.0	0.5	0.5	*nfxB*	Nonsynonymous mutation (R42H)
cipR2	4.0	0.5	0.5	*nfxB–[morA]*	Δ4,649 bp deletion
cipR3	2.0	0.5	0.5	*nfxB*	Nonsynonymous mutation (A33P)
cefR1	0.25	8.0	0.5	*dacB*	Δ5 bp deletion
cefR2	0.25	4.0	0.5	*[PA3046]–[dacB]*	Δ1,249 bp deletion
cefR3	0.25	8.0	0.5	*dacB*	Nonsynonymous mutation (A343E)
merR1	0.5	2.0	8.0	*[phoQ]–[PA1181]-[PA1182]*	Δ4,122 bp deletion
				*nalD*	Nonsynonymous mutation (T11N)
merR2	1	2.0	8.0	*mexR*	Δ12 bp deletion
				*phoQ*	Nonsynonymous mutation (V382G)
merR3	0.5	1.0	8.0	*oprD*	Δ11 bp deletion
				*phoQ*	Nonsynonymous mutation (V260G)
				*nalD*	Nonsynonymous mutation (T11P)

**Figure 2.**
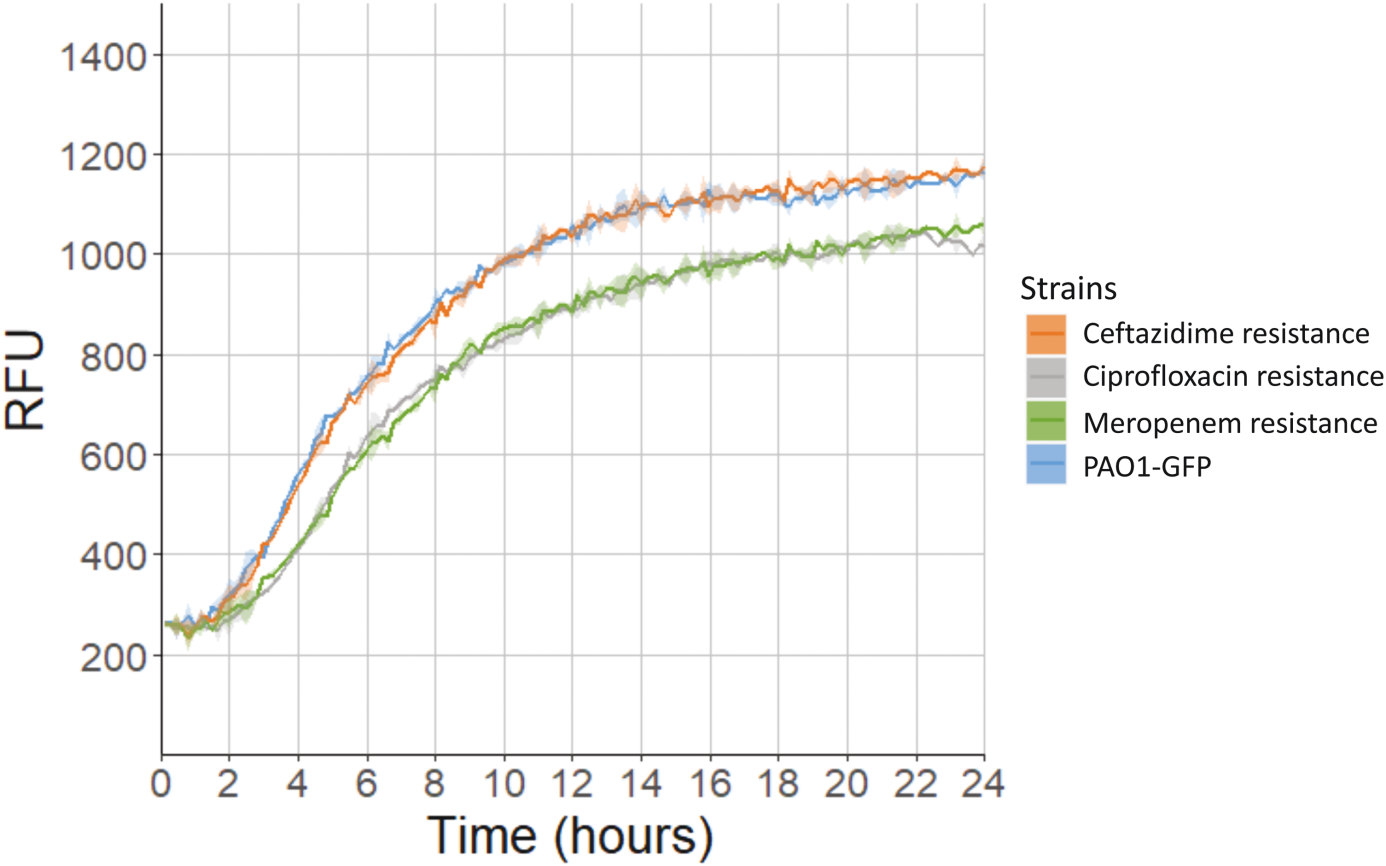
Growth rate assays of the ceftazidime-, ciprofloxacin-, and meropenem-resistant strains compared to PAO1-GFP in tryptic soy broth in the absence of respiratory microbes. Growth was measured as relative fluorescence units (RFU) in the GFP channel over a period of 24 hr, and data were plotted as mean (three biological replicates with two technical replicates for each resistance phenotype) and standard error of the mean (shaded area) using the Growthcurver package in R (Sprouffske & Wagner, 2016).

Selection on ceftazidime was associated with the acquisition of mutations in *dacB* that are known to cause the overexpression of the AmpC β-lactamase and increased resistance to ceftazidime (Aguilera Rossi et al., 2016). The meropenem-resistant mutants (merR1–merR3) were selected through a short 3-day passage experiment of increasing meropenem concentration. This is because no spontaneous resistant mutants were able to grow from overnight culture plating at the clinical breakpoint, and these strains had a larger number of mutations than the ciprofloxacin- and ceftazidime-resistant mutants (Figure 1). The genetic basis of meropenem resistance in *P. aeruginosa* is complex and clinical resistance levels require two or more mutations (Cabot et al., 2016; López-Causapé et al., 2018), this is reflected in the increased complexity of the meropenem-resistant mutants that we isolated (Table 1). Meropenem-resistant mutants carried mutations in genes such as *phoQ*, *mexR*, and *nalD* that are known to be associated with elevated expression of multidrug efflux pumps (Miryala et al., 2019; Morita et al., 2006). One of the mutants (merR3) also carried a deletion in *oprD*, which encodes the outer porin OprD that can serve as a channel to let meropenem into the cell (Cabot et al., 2016; Pai et al., 2001). These mutations in genes involved in multidrug efflux pump regulation likely explain why the meropenem-resistant mutants had increased resistance to both ceftazidime and ciprofloxacin too (Table 1). The ciprofloxacin and meropenem-resistant mutants demonstrated decreased growth compared to PAO1-GFP in rich media (as measured by RFU over time), whereas growth of strains with ceftazidime resistance was more comparable to PAO1-GFP (Figure 2).

### Invasion of *P. aeruginosa*-resistant mutants into pre-established respiratory microbe cultures

**Figure 3.**
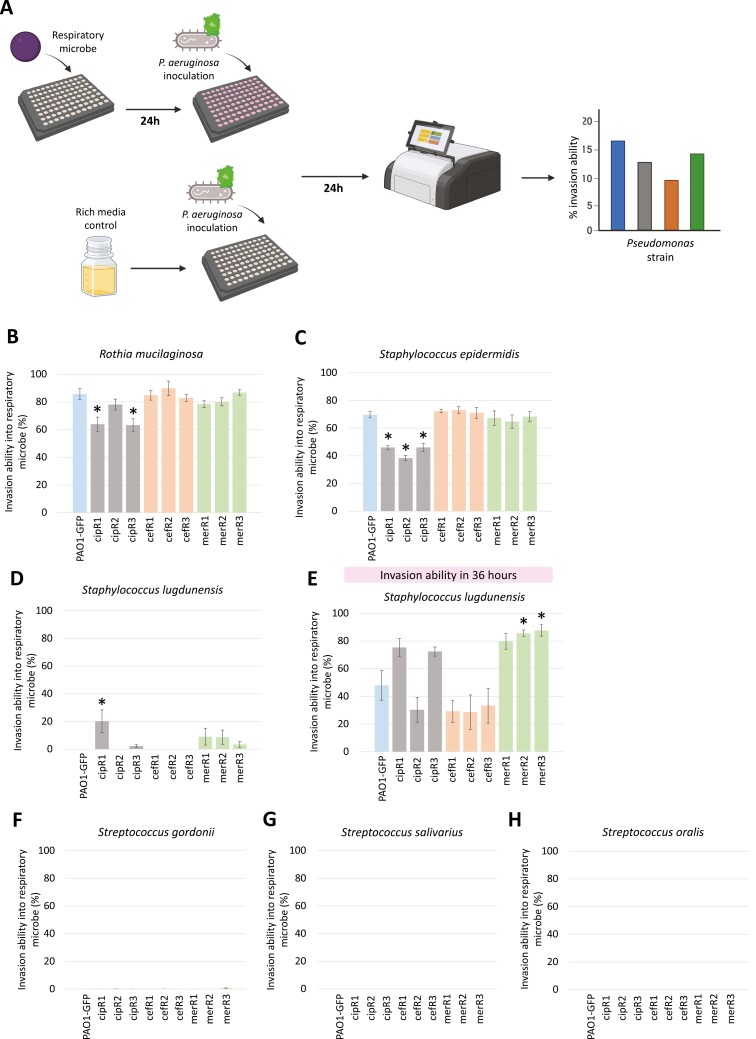
Schematic of invasion assay method (A) and invasion of *P. aeruginosa* into respiratory microbiome strains in 24 hr (B, C, D, F, G, and H) and 36 hr (E). Each bar chart shows invasion ability measured as percentage RFU compared to the media control at that same timepoint (y-axis), for invasion into pre-established cultures of: (B) *R. mucilaginosa*, (C) *S. epidermidis*, (D) *S. lugdunensis* (24 hr), (E) *S. lugdunensis* (36 hr), (F) *S. gordonii*, (G) *S. salivarius*, and (H) *S. oralis*. Values obtained in *S. gordonii*, *S. salivarius*, and *S. oralis* were considered negligible and below the limits of the assay (below 2%). Differences in invasion ability between the *P. aeruginosa* antibiotic-resistant strains and PAO1-GFP were assessed using a one-way ANOVA followed by a post hoc Dunnett’s test, and significance (Dunnett’s test: *p* < 0.05) is indicated by an asterisk (*). Bar charts show the mean of six biological replicates ± standard error of the mean.

We measured the ability of the *P. aeruginosa*-resistant mutants to invade the pre-established cultures of the respiratory microbiome strains by measuring GFP at 24 hr after *P. aeruginosa* inoculation (Figure 3A). We calculated invasion ability as the growth of *P. aeruginosa* in the presence of the respiratory microbe compared to the paired growth of *P. aeruginosa* in tryptic soy broth (in the absence of a respiratory microbe). Invasion assay results are shown as percentage growth in respiratory microbe invasion compared to the media control (Figure 3A). Prior to these assays in which endpoint measurements were taken, invasion was first continually monitored in a BioTek Synergy 2 microplate reader (Supplementary Figure S2).

All respiratory microbe cultures provided some degree of resistance to *P. aeruginosa* growth, and this effect was particularly strong for the *Streptococcus* species and *S. lugdunensis* (Figure 3). *Pseudomonas aeruginosa* was best able to invade *R. mucilaginosa*, with PAO1-GFP reaching ~86% (±4%) of the RFU reached in the absence of *R. mucilaginosa* (Figure 3B). Second to this was *S. epidermidis*, with PAO1-GFP reaching ~70% (±2%) of the RFU reached in the absence of *S. epidermidis* (Figure 3C). The ceftazidime- and meropenem-resistant mutants showed no change in invasion ability into *R. mucilaginosa* and *S. epidermidis* (Figure 3B, C). However, the ciprofloxacin-resistant mutants had an impaired invasion ability in both *R. mucilaginosa* (2/3: cipR1, cipR3) and *S. epidermidis* (3/3: cipR1, cipR2, and cipR3) (Figure 3B, C), suggesting a clear cost to resistance in this context.

The ceftazidime-resistant mutants showed no change in invasion ability into cultures of *S. lugdunensis*, but ciprofloxacin- and meropenem-resistant mutants demonstrated an increased invasion ability (Figure 3D). While the ancestral PAO1-GFP strain was unable to invade *S. lugdunensis* in 24 hr, five of the resistant mutants (cipR1, cipR3, merR1, merR2, and merR3) were able to grow in this same timeframe (defined as at least 2% of RFU in media in absence of *S. lugdunensis*) (Figure 3D). The invasion of the resistant mutants into *S. lugdunensis* was near the lower limit of detection (Figure 3D). We extended this assay to 36 hr. In 36 hr, PAO1-GFP was now able to invade the pre-established *S. lugdunensis* culture (Figure 3E). The five resistant mutants (cipR1, cipR3, merR1, merR2, and merR3) demonstrated a trend toward increased invasion ability (Figure 3E), suggesting a benefit to resistance in this context. It should be noted that statistical significance as assessed by a one-way ANOVA followed by a post hoc Dunnett’s test (*p* < 0.05) was only observed for cipR1 at 24 hr and merR1/merR2 at 36 hr (Figure 3E).

Finally, the *Streptococcus* spp. (*S. gordonii*, *S. salivarius*, and *S. oralis*) showed strong resistance to *P. aeruginosa* growth, which was maintained regardless of the antibiotic resistance genotype (Figure 3F, G, H). *Pseudomonas aeruginosa* was unable to invade in 24 hr, and this effect was preserved when we extended the assay to 36 hr (Supplementary Figure S3). Across these assays (Figure 3), changes to invasion ability were observed for the ciprofloxacin- and meropenem-resistant mutants, but the three ceftazidime-resistant mutants (cefR1-cefR3) associated with mutations in *dacB* were not found to alter invasion into any of the six respiratory microbes.

### Invasion of *P. aeruginosa* clinical isolates into *S. lugdunensis*

**Figure 4.**
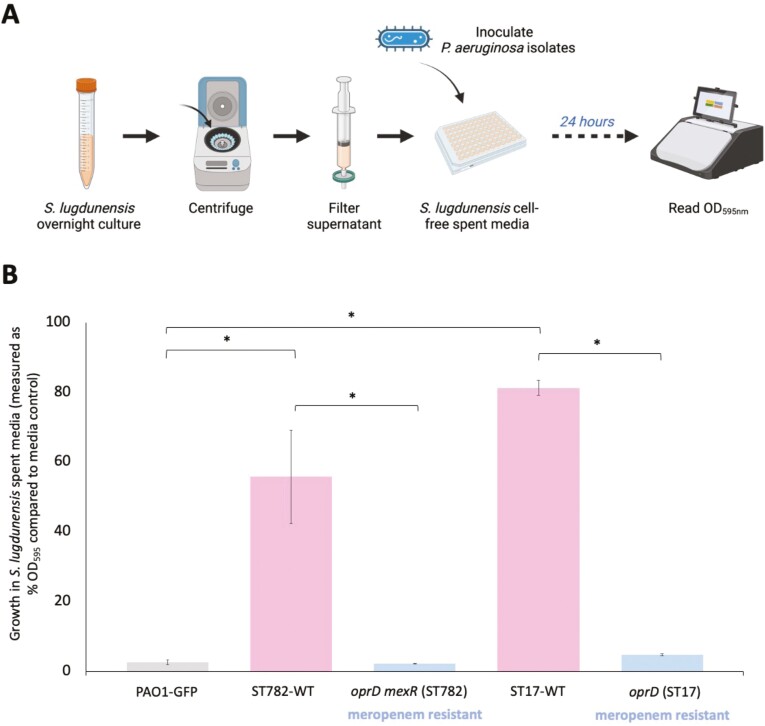
(A) Schematic of *S. lugdunensis* spent media assay. (B) Growth of PAO1-GFP and clinical *P. aeruginosa* isolates in *S. lugdunensis* spent media, measured as percentage OD_595_ in *S. lugdunensis* spent media compared to the media control at 24 hr. For each clinical *P. aeruginosa* genotype, three biological replicates of three isolates were measured and the values are plotted as mean ± standard error of the mean. For PAO1-GFP, three biological replicates were measured. One tailed unpaired *t*-tests were carried out to compare growth in *S. lugdunensis* spent media between PAO1-GFP and the two clinical WT strains (ST782-WT, ST17-WT) and to compare between each resistant mutant to its WT background. Significance as *p* < 0.05 is indicated by an asterisk (*).

Invasion of *P. aeruginosa* into *S. lugdunensis* was an interesting example of where antibiotic resistance seemed to improve the ability of *P. aeruginosa* to invade (Figure 3D, E). To better characterize this interaction, we repeated these invasion assays using isolates from two patients in which meropenem resistance evolved during *P. aeruginosa* infection due to the acquisition of de novo mutations (Supplementary Table S1; Wheatley et al., 2021, 2022). These isolates were obtained from two hospitalized patients that were previously recruited as part of an observational trial into resistance evolution during *P. aeruginosa* infections in intensive care units (Diaz Caballero et al., 2023; Paling et al., 2017), and are described in detail here: (Wheatley et al., 2021, 2022). In brief, one patient was colonized by Sequence Type (ST) 782 *P. aeruginosa* (ST782-WT), and increased resistance to meropenem evolved via mutation to *oprD* and *mexR* (*oprD mexR* ST782) (Wheatley et al., 2022). The second patient was colonized by ST17 *P. aeruginosa* (ST17-WT), and increased resistance to meropenem evolved via mutation to *oprD* (*oprD* ST17) (Wheatley et al., 2021). These assays were carried out as spent media assays in which *P. aeruginosa* invasion ability was measured using OD_595_ reads as these clinical isolates were not GFP-tagged (Figure 4A), and PAO1-GFP was tested in parallel in this assay.

PAO1-GFP was unable to grow in *S. lugdunensis* spent media over 24 hr (Figure 4B). Duplicating the effect observed in *S. lugdunensis* culture (Figure 3D) and indicating that the resistance to *P. aeruginosa* growth mediated by *S. lugdunensis* is independent of *P. aeruginosa* detection or contact. However, this inhibitory effect was weak for the clinical isolates ST17-WT and ST782-WT, which were able to grow to ~80% and ~55% of the OD_595nm_ reached in the media controls, respectively (Figure 4B). Mutations in ST17 (*oprD*) and ST782 (*oprD mexR*) isolates that increased meropenem resistance during infection were associated with strongly reduced growth in *S. lugdunensis* spent media (Figure 4B), which is opposite to the effect of meropenem resistance observed in PAO1-GFP. This opposing outcome could be due to either the impact of genetic background or it could suggest that the mutations to *oprD* and possibly *mexR* too did not contribute to the improved invasion ability of the meropenem-resistant PAO1-GFP mutants (Figure 3).

We hypothesized that the resistance of *S. lugdunensis* to PAO1-GFP growth observed in the invasion assays (Figure 3D) could be attributable to the presence of an inhibitory molecule secreted by *S. lugdunensis* that is active against PAO1-GFP. In support of this hypothesis, resistance to PAO1-GFP growth was also observed in the spend media assay (Figure 4A). To test this hypothesis, we carried out a supernatant inhibition plating assay where we grew PAO1-GFP on agar plates in the presence of a filter disc inoculated with either *S. lugdunensis* supernatant or ddH_2_O as a negative control. We observed no zone of inhibition compared to the negative control (Supplementary Figure S4A, B). We repeated this assay spotting the supernatant directly onto the plate, in case the inhibition was mediated by a molecule that was freeze-sensitive, and observed no growth inhibition (Supplementary Figure S4C, D). From this we concluded that the resistance of *S. lugdunensis* to *P. aeruginosa* invasion is more likely mediated by depletion of metabolites required by *P. aeruginosa* during *S. lugdunensis* growth, rather than direct inhibition via secreted inhibitory molecules.

## Discussion

The emergence and spread of antibiotic resistance in bacterial pathogens poses a serious threat to public health (Ikuta et al., 2022; Murray et al., 2022). The key finding from our work is that antibiotic resistance can change the ability of a bacterial pathogen to invade pre-established cultures of bacteria from the respiratory microbiome. This can occur in both positive and negative directions, which are *P. aeruginosa* genotype and respiratory microbe identity dependent. We show how the cost of mutations that provide enhanced antibiotic resistance in *P. aeruginosa* can crucially depend on community context, and this has clear relevance to our ability to predict how resistance will spread in bacterial pathogen populations.

While *P. aeruginosa* was best able to invade *R. mucilaginosa* and *S. epidermidis* cultures, the ciprofloxacin-resistant *nfxB* mutants had an impaired invasion ability into both *R. mucilaginosa* and *S. epidermidis* (Figure 3B, C). This is in line with work by others demonstrating that the presence of a microbial community can increase the fitness costs associated with resistance in *Escherichia coli* (Baumgartner et al., 2020; Klümper et al., 2019)*. Rothia mucilaginosa* can help facilitate *P. aeruginosa* growth via cross-feeding (Gao et al., 2018), which may help explain the high growth of *P. aeruginosa* in *R. mucilaginosa* culture invasion. We hypothesized that the reduced invasion ability of the ciprofloxacin-resistant *nfxB* mutants could be due to an increased efflux of *R. mucilaginosa*-produced metabolites that support *P. aeruginosa* growth via the MexCD-OprJ efflux pump, which is negatively regulated by NfxB (Pang et al., 2019; Poole et al., 1996). We speculate that a similar hypothetical mechanism could mediate this interaction with *S. epidermidis*. *Pseudomonas aeruginosa* can uptake *Staphylococcus aureus* secreted metabolites including acetoin that can be catabolized by *P. aeruginosa* (Camus et al., 2020; Zarrella & Khare, 2022) and *S. epidermidis* is also capable of acetoin production (Hara et al., 2014).

Strikingly, we found that certain *P. aeruginosa-*resistant mutants (cipR1, cipR3, merR1, merR2, and merR3) were able to invade *S. lugdunensis* in a period of 24 hr, when the ancestral strain (PAO1-GFP) could not (Figure 3D). These resistant mutants carried mutations in genes such as *nfxB*, *mexR*, *nalD*, and *phoQ* that are known to be associated with elevated expression of multidrug efflux pumps (e.g., MexCD-OprJ and MexAB-OprM) (Table 1) (Miryala et al., 2019; Morita et al., 2006). One of the mutants (merR3) also carried a deletion in *oprD*, which encodes the outer porin OprD, a well-characterized channel for meropenem entry into the cell (Cabot et al., 2016; Pai et al., 2001).

The mutations observed suggested that upregulation of multidrug efflux pumps (e.g., MexCD-OprJ and MexAB-OprM) is beneficial for invasion into *S. lugdunensis.* This led us to hypothesize that *S. lugdunensis* is secreting an inhibitory molecule that can be exported by these pumps, but when we tested this hypothesis using a supernatant plating assay no significant zone of inhibition was observed (Supplementary Figure S4C, D). Alternatively, it is possible that *S. lugdunensis-*mediated resistance to *P. aeruginosa* growth may be driven by depletion of metabolites required by *P. aeruginosa*. Under this model, the growth of *S. lugdunensis* restricts *P. aeruginosa* indirectly via the consumption of nutrients needed for *P. aeruginosa* growth in this environment. The importance of “nutrient blocking” in providing resistance to pathogen invasion in the microbiome has been demonstrated in recent work (Spragge et al., 2023). Furthermore, it has been shown that the ability of multidrug-resistant *E. coli* lineages to displace commensal *E. coli* in the gut microbiome can be facilitated by encoding a high genetic diversity of carbohydrate metabolism genes, which can help provide a competitive advantage to invade in these environments (Connor et al., 2023). While it is unclear exactly how the upregulation of multidrug efflux pumps (e.g., MexCD-OprJ and MexAB-OprM) in PAO1-GFP may be beneficial for invasion into *S. lugdunensis,* overexpression of these efflux pumps has been linked to multiple effects (Alcalde-Rico et al., 2018; Alcalde‐Rico et al., 2020; Linares et al., 2005; Maseda et al., 2004; Peng et al., 2017).

The method used for our main analysis, comparing many mutants from one background (PAO1), provided the cleanest way to look at the impact of multiple mutations. Comparing mutant–ancestor pairs from different backgrounds would be an alternative way to carry out this type of experiment to also look at the impact of genetic background. When we investigated the invasion ability into *S. lugdunensis* of clinical *P. aeruginosa* isolates from different sequence types (ST17 and ST782) (Figure 4), we found that the ancestral isolates were able to grow significantly better in the cell-free spent media of *S. lugdunensis* compared to PAO1 (Figure 4B), but that isolates with *oprD* or *oprD mexR* mutations showed a dramatically reduced invasion ability (Figure 4B). This contrasts with what was observed with meropenem resistance in PAO1. Given that OprD is a porin, we speculate that loss of OprD may hinder the ability of clinical isolates to acquire metabolites that are effectively depleted by *S. lugdunensis.* This speculation lends support to a role of nutrients in helping *S. lugdunensis* resistance to *P. aeruginosa*. This differing invasion phenotype into *S. lugdunensis* observed could be due to the importance of genetic background in determining the outcome of this interaction. Or, suggest that mutation to *oprD* did not contribute to the improved invasion ability of PAO1 merR3. It should be noted that the background strain used for the main analysis (PAO1-GFP) carried a gentamicin resistance cassette, integrated in strain construction (Vogwill et al., 2016). There is insufficient evidence that *P. aeruginosa* is a good target for gentamicin therapy (EUCAST, 2024), and gentamicin resistance is common in natural *P. aeruginosa* clones (Diaz Caballero et al., 2023; Jansen et al., 2016). *Pseudomonas aeruginosa* strains can vary significantly in their carriage of acquired resistance mechanisms (Diaz Caballero et al., 2023; Sastre-Femenia et al., 2023), and future work will look to further explore the impact of resistance mutations in different genetic backgrounds, which may vary in carriage of additional resistance mechanisms and standing phenotypic resistance.

Finally, we identified three *Streptococcus* species (*S. gordonii*, *S. salivarius*, and *S. oralis*) that *P. aeruginosa* was unable to invade, regardless of resistance genotype (Figure 3F, G, H). These streptococcal species are known for their ability to inhibit *P. aeruginosa* through a variety of mechanisms (Stoner et al., 2023; Tunçer & Karaçam, 2020; Whiley et al., 2015), including media acidification (Abranches et al., 2018), hydrogen peroxide production (Kreth et al., 2008), and through production of a variety of bacteriocin-like inhibitory substances (Wescombe et al., 2009). *S. salivarius* strains have been developed for oral probiotic applications that are commercially available (Santagati et al., 2012). Our research adds to this body of literature and demonstrates that successful inhibition of *P. aeruginosa* occurs for a variety of resistance genotypes that have been generated by selection on important antipseudomonal drugs.

## Conclusions

In summary, we find that specific antibiotic resistance mutations can change the ability of *P. aeruginosa* to invade pre-established cultures of microbes of the respiratory microbiome. This occurs in both positive and negative directions, which are resistance genotype and respiratory microbe identity dependent. Our work demonstrates how the cost of mutations that provide enhanced antibiotic resistance in *P. aeruginosa* can crucially depend on community context. For example, ciprofloxacin-resistant strains associated with mutations in *nfxB* showed an improved invasion ability into *S. lugdunensis,* but a reduced invasion ability into *S. epidermidis* and *R. mucilaginosa.* On the other hand, ceftazidime-resistant strains associated with mutations in *dacB* were not found to alter invasion into any of the six respiratory microbes, and *Streptococcus* species provided the strongest resistance to *P. aeruginosa* invasion, which was maintained regardless of antibiotic resistance genotype. Looking to the future, we argue that understanding this interface between antibiotic resistance and species interactions has clear significance to our ability to predict the spread of antibiotic resistance in bacterial pathogens, and that attempts to manipulate the respiratory microbiome should focus on promoting the growth of commensals that can provide robust inhibition of both wildtype and resistant mutant strains.

## Supplementary material

Supplementary material is available online at *Evolution Letters*.

qrae030_suppl_Supplementary_Table_S1

qrae030_suppl_Supplementary_Table_S2

qrae030_suppl_Supplementary_Figures

## Data Availability

All sequencing data generated in this study can be found at: (http://doi.org/10.5281/zenodo.11084333). All *P. aeruginosa* isolates generated in this study can be obtained via the corresponding author for research use.
