# Supplementary material for: Antibiotic resistance alters the ability of Pseudomonas aeruginosa to invade bacteria from the respiratory microbiome
Source: Evol Lett. 2024 Jun 30;8(5):735–47. doi: 10.1093/evlett/qrae030 (PMC11424078; doi:10.1093/evlett/qrae030)

Supplementary Information

Supplementary Figure 1. Plate layout for invasion assays.

The respiratory microbe cultures were arranged into a 96-well plate such that six replicate cultures were present in each of the 10 inner well columns of the plate, as is shown below of the example of 'SE':

	1	2	3	4	5	6	7	8	9	10	11	12
A												
B	rep 1	SE-1	SE-1	SE-1	SE-1	SE-1	SE-1	SE-1	SE-1	SE-1	SE-1	
C	rep 2	SE-2	SE-2	SE-2	SE-2	SE-2	SE-2	SE-2	SE-2	SE-2	SE-2	
D	rep 3	SE-3	SE-3	SE-3	SE-3	SE-3	SE-3	SE-3	SE-3	SE-3	SE-3	
E	rep 4	SE-4	SE-4	SE-4	SE-4	SE-4	SE-4	SE-4	SE-4	SE-4	SE-4	
F	rep 5	SE-5	SE-5	SE-5	SE-5	SE-5	SE-5	SE-5	SE-5	SE-5	SE-5	
G	rep 6	SE-6	SE-6	SE-6	SE-6	SE-6	SE-6	SE-6	SE-6	SE-6	SE-6	
H												

The *P. aeruginosa* strains were then inoculated into this plate for six replicates of each strain as shown below:

	1	2	3	4	5	6	7	8	9	10	11	12
A												
B	rep 1	PAO1-GFP-1	cipR1-1	cipR2-1	cipR3-1	cefR1-1	cefR2-1	cefR3-1	merR1-1	merR2-1	merR3-1	
C	rep 2	PAO1-GFP-2	cipR1-2	cipR2-2	cipR3-2	cefR1-2	cefR2-2	cefR3-2	merR1-2	merR2-2	merR3-2	
D	rep 3	PAO1-GFP-3	cipR1-3	cipR2-3	cipR3-3	cefR1-3	cefR2-3	cefR3-3	merR1-3	merR2-3	merR3-3	
E	rep 4	PAO1-GFP-4	cipR1-4	cipR2-4	cipR3-4	cefR1-4	cefR2-4	cefR3-4	merR1-4	merR2-4	merR3-4	
F	rep 5	PAO1-GFP-5	cipR1-5	cipR2-5	cipR3-5	cefR1-5	cefR2-5	cefR3-5	merR1-5	merR2-5	merR3-5	
G	rep 6	PAO1-GFP-6	cipR1-6	cipR2-6	cipR3-6	cefR1-6	cefR2-6	cefR3-6	merR1-6	merR2-6	merR3-6	
H												

Key

(orange)	Outer wells filled with media
(blue)	Inner wells used for invasion assay

Supplementary Figure 2. Continuous monitoring of invasion assays over 24 hours in (A) *Rothia mucilaginosa*, (B) *Staphylococcus epidermidis*, (C) *Staphylococcus lugdunensis* to produce pilot growth curves for illustrative purposes (PAO1-GFP = blue, cipR1-R3 = gray, cefR1-R3 = orange, merR1-R3 = green).

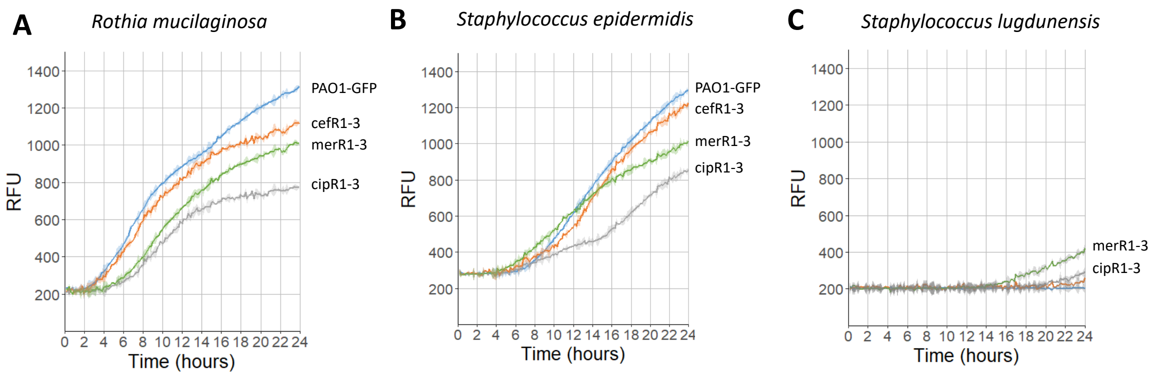

Supplementary Figure 3. Invasion of *P. aeruginosa* strains into *Streptococcus* species over 36 hours.

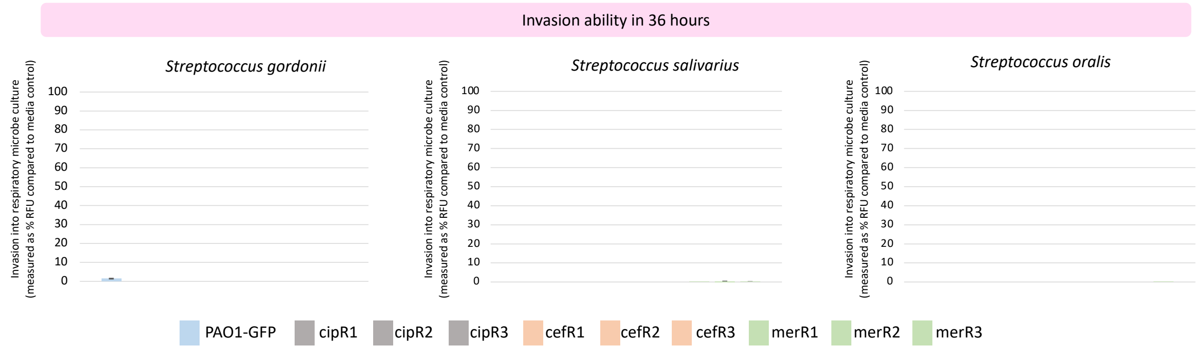

Supplementary Figure 4. Plate images for supernatant inhibition assay. (A) *S. lugdunensis* spent media or ddH$_2$0 filter disc assay. Image shows x1000 dilution plating of overnight PAO1-GFP culture, taken with iBright CL1000 imaging. (B) *S. lugdunensis* spent media or ddH$_2$0 filter disc assay. Image shows x1000 dilution plating of overnight PAO1-GFP culture, taken with blue light imaging. (C) *S. lugdunensis* spent media or ddH$_2$0 supernatant spotting assay. Image shows x1000 dilution plating of overnight PAO1-GFP culture, taken with iBright CL1000 imaging. (D) *S. lugdunensis* spent media or ddH$_2$0 supernatant spotting assay. Image shows x1000 dilution plating of overnight PAO1-GFP culture, taken with blue light imaging. No zone of inhibition (compared to the negative ddH$_2$0 control) was observed in any of the replicates.

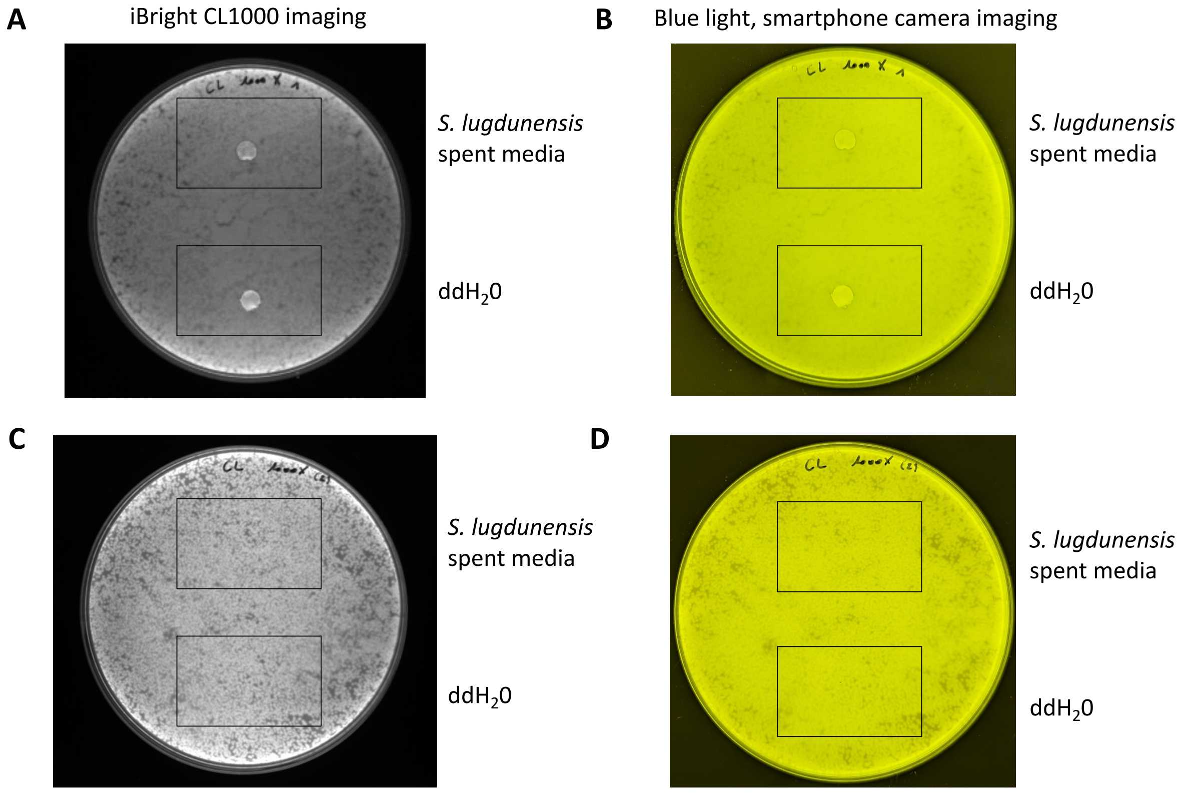

Supplement: qrae030_suppl_Supplementary_Figures [file qrae030_suppl_supplementary_figures.pdf]
